# A SYSTEMATIC REVIEW ON CAREGIVING FOR ABUSIVE FAMILY MEMBERS

**DOI:** 10.1093/geroni/igz038.2490

**Published:** 2019-11-08

**Authors:** Qiyi Zhang

**Affiliations:** University of Pittsburgh, School of Social Work, Pittsburgh, Pennsylvania, United States

## Abstract

While elder abuse perpetrated by caregivers has raised significant attention in the past 30 years, the well being of and support for caregivers of abusive or previously abusive family members remains little known. This population is large in scope, but greatly neglected in the scientific investigation of elder abuse or domestic violence. This systematic review aims to summarize conceptual discussions and empirical findings from existing peer-reviewed articles to shed light on how to understand, identify, and provide support for this group. I performed a search using MEDLINE, PsycINFO, Cochrane, CINAHL, EMBASE, Ageline, and Social Work Abstract, and examined reference lists of identified articles in the stage of full-text screening. Targeted articles are written in English, peer-reviewed, published between 1989 to 2019, and contribute significant knowledge to the study aims. The search concluded with four conceptual articles and six empirical articles. This review reveals a change in approaches in understanding the living situation of maltreated caregivers: previously an indicator of poor caregiving relationship for predicting caregiving outcomes, now it is increasingly acknowledged as a vulnerable group in need of special attention and support. Empirical studies showed that a significant percentage of caregivers have a history of being abused, and they suffered from worse mental health and responded more poorly to certain coping mechanisms than caregivers without those experiences. Stress and self-esteem were found to be possible mediators. Competing conceptual frameworks on this issue include: 1) abuse versus interpersonal violence; 2) life course perspective; 3) communal model versus exchange model of caregiving.

